# Interpersonal cognitive distortions as mediators of the relationship between psychological distress and nomophobia in adolescents: a cross-sectional mediation analysis

**DOI:** 10.3389/fpsyg.2026.1810935

**Published:** 2026-09-07

**Authors:** Sarah Javed, Heena Parveen, Salma Kaneez, Ayoob Lone, Fahad Alwadani, Nawaf Al Khashram

**Affiliations:** 1 Department of Psychology, Aligarh Muslim University, Aligarh, Uttar Pradesh, India; 2 Women’s College, Aligarh Muslim University, Aligarh, Uttar Pradesh, India; 3 Department of Clinical Neurosciences, College of Medicine, King Faisal University, AlAhsa, Saudi Arabia; 4 Department of Surgery, College of Medicine, King Faisal University, AlAhsa, Saudi Arabia; 5 Department of Biomedical Sciences, College of Medicine, King Faisal University, AlAhsa, Saudi Arabia

**Keywords:** adolescents, interpersonal cognitive distortions, nomophobia, psychological distress, smartphone

## Abstract

The increasing reliance on smartphones for emotional relief and social connectivity has contributed to the rise of nomophobia. The present study examined interpersonal cognitive distortions (ICDs) as mediators of the relationship between psychological distress and nomophobia in adolescents using a cross-sectional mediation design. A sample of 500 adolescents completed standardized measures assessing psychological distress (DASS-21), nomophobia (NMP-Q), and interpersonal cognitive distortions. To examine the hypothesized mechanisms through which psychological distress influences nomophobia, a parallel mediation analysis was conducted using PROCESS Macro (Model 4). The significance level was set at <0.05 throughout the analysis. Findings from parallel mediation analysis revealed that psychological distress significantly predicts nomophobia, both directly and indirectly through two dimensions of ICDs: interpersonal rejection and unrealistic relationship expectations. However, interpersonal misperception did not mediate this relationship. Findings suggest that emotionally distressed individuals may turn to smartphones as a maladaptive coping strategy to avoid social discomfort and seek digital reassurance. This pattern fosters a self-reinforcing cycle of avoidance, cognitive distortion, and digital dependency. The study contributes to a nuanced understanding of nomophobia’s development and highlights the importance of addressing both emotional and cognitive vulnerabilities in interventions aimed at reducing problematic smartphone use.

## Introduction

The increasing reliance on smartphones has significantly altered modern communication, social interactions, family dynamics, and emotional regulation (McDaniel, 2021; Parent and Shapka, 2020). While these devices provide opportunities for connectivity, their excessive use has also led to psychological concerns, most notably nomophobia, the irrational fear of being without a mobile device (Yildirim and Correia, 2015). Research has consistently demonstrated associations between nomophobia and psychological vulnerabilities, yet the cognitive pathways underlying these associations remain underexplored (Elhai et al., 2019; Alhusseini et al., 2025).

Psychological distress represents a significant form of vulnerability. It refers to a state of emotional discomfort or internal suffering that emerges when individuals encounter difficult or stressful life situations. This condition is marked by a combination of nonspecific psychological and physical symptoms, such as persistent sadness, heightened anxiety, irritability, restlessness, or bodily complaints like fatigue and aches usually triggered by stressful life circumstances (Bessaha, 2015). Importantly, psychological distress is broader than, and not identical to, anxiety or depression; it encompasses these symptoms as components of a wider state of impaired wellbeing (Zhu et al., 2022). Distressed individuals often show withdrawal, emotional dysregulation, and avoidance of interpersonal contact, leading them to seek alternative coping strategies such as digital engagement (Cai et al., 2023; Brouwer et al., 2021). Over time, reliance on smartphones for relief reinforces avoidance behaviors and may worsen distress through maladaptive dependence (Elhai et al., 2020).

Studies suggest that smartphone separation amplifies fear and anxiety, particularly among individuals already experiencing psychological distress (Alhusseini et al., 2025; Gezgin et al., 2018; Santl et al., 2022). However, in line with the focus of the present research, the emphasis is not that nomophobia causes distress, but that distress predisposes individuals to nomophobia. In this sense, adolescents in distress may become more likely to depend on smartphones as a coping mechanism, which ultimately fosters pathological fear of disconnection (Abdoli et al., 2023).

Recent research further situates nomophobia within the broader context of digital dependence and digital wellbeing. Durmuş Sarıkahya et al. (2026) reported that nomophobia mediated the association between digital game addiction and fatigue among university students, while Netless phobia moderated this relationship. These findings suggest that nomophobia may function as an important mechanism through which excessive digital engagement contributes to adverse psychological and functional outcomes. Thus, nomophobia may be understood not only as an isolated fear of smartphone disconnection but also as part of a broader pattern of maladaptive digital dependence. Complementing this evidence, Özbay et al. (2026) found that university students who engaged in digital detox practices had lower levels of nomophobia and Netless phobia and higher academic motivation, self-efficacy, and academic performance. Together, these findings highlight the importance of examining nomophobia within a broader digital wellbeing framework in which emotional vulnerability, maladaptive cognitions, and patterns of digital engagement may interact. However, these studies primarily examined university students and focused on digital game addiction, fatigue, or digital detox, leaving the cognitive mechanisms linking psychological distress to nomophobia among adolescents insufficiently understood. The present study addresses this gap by examining interpersonal cognitive distortions as potential mediating mechanisms between psychological distress and nomophobia.

A key mechanism that may help explain this pathway lies in interpersonal cognitive distortions. ICDs are irrational and maladaptive beliefs about social relationships, including expectations of rejection, misinterpretation of social cues, and unrealistic standards for relationships (Hamamcı and Büyüköztürk, 2004). These distortions are known to contribute to social anxiety, rejection sensitivity, and avoidance of face-to-face interactions (O’Day and Heimberg, 2021; Li et al., 2023). When distressed adolescents perceive heightened rejection or hold unrealistic expectations, they may increasingly turn to smartphones to obtain controlled, predictable, and safer social interactions (Lu and Yeo, 2015). Although previous studies have used mediation and moderation models to examine psychological and interpersonal mechanisms underlying nomophobia, the mechanisms investigated have generally involved constructs such as attachment, loneliness, online social interaction, social support, personality, and self-control. For example, Sun et al. (2024) identified attachment and loneliness as mediators in the relationship between neuroticism and nomophobia, while Khan et al. (2025) examined online social interaction as a mediator and social support as a moderator in the association between loneliness and nomophobia. Similarly, previous research has examined attachment-related mechanisms and other psychological vulnerabilities in explaining nomophobia (Karaoglan Yilmaz et al., 2024; Thakur, 2024). These studies provide important evidence that nomophobia may develop through multiple affective, interpersonal, and behavioral pathways. However, they have not directly examined whether specific interpersonal cognitive distortions serve as mediating mechanisms linking psychological distress to nomophobia.

Some scholars have suggested that nomophobia itself may represent a form of interpersonal cognitive distortion, as it reflects maladaptive fears of disconnection and loss of validation from others (Enez, 2021). While acknowledging this overlap, the present study distinguishes ICDs as broader antecedent cognitions that predispose individuals to nomophobia rather than being identical constructs. Specifically, the ICDS framework identifies three validated dimensions: interpersonal rejection, unrealistic relationship expectations, and interpersonal misperception: that are particularly relevant in adolescence (Hamamcı and Büyüköztürk, 2004). Rejection reflects hypersensitivity to exclusion, unrealistic expectations involve rigid standards of reciprocity, and misperception entails biased interpretations of others’ behaviors. These patterns align with developmental vulnerabilities of adolescence, a period marked by peer sensitivity and unstable self-concepts (Twenge and Campbell, 2018).

To situate this relationship theoretically, the current study integrates three perspectives. The Uses and Gratifications Theory (UGT; Katz et al., 1974) explains why individuals actively seek smartphones to satisfy unmet cognitive, affective, or social needs. The Compensatory Internet Use Theory (CIUT; Kardefelt-Winther, 2014) extends this by emphasizing that digital use often functions as a coping strategy to escape negative emotions or compensate for offline difficulties. Within this framework, ICDs can be understood as amplifiers of interpersonal motives, for instance, rejection sensitivity may increase the drive to maintain constant online connection. The Matthew Effect (Merton, 1968) offers a broader lens to understand how pre-existing disadvantages, such as distress, compound over time, thereby magnifying vulnerability to maladaptive smartphone reliance. In this study, Matthew Effect is not used to suggest nomophobia worsens distress, but to highlight how cumulative vulnerabilities can reinforce cycles of digital dependence.

Taken together, the existing literature indicates that mediation and moderation approaches have successfully identified several pathways associated with nomophobia, but the focus has predominantly been on affective, interpersonal, personality-related, and behavioral factors. The present study advances this literature by examining a distinct interpersonal-cognitive pathway. Specifically, rather than focusing on loneliness, attachment, social interaction, or self-regulation, the current model proposes that psychological distress may be associated with nomophobia through maladaptive beliefs and expectations concerning interpersonal relationships. Furthermore, the parallel mediation model allows the three dimensions of interpersonal cognitive distortions—interpersonal rejection, unrealistic relationship expectations, and interpersonal misperception—to be examined simultaneously. This distinction is important because different forms of interpersonal cognition may not contribute equally to nomophobia. Therefore, the novelty of the present study lies in identifying specific interpersonal cognitive mechanisms through which psychological distress may be associated with nomophobia among adolescents, rather than simply applying another mediation model to nomophobia.

**Figure 1:**
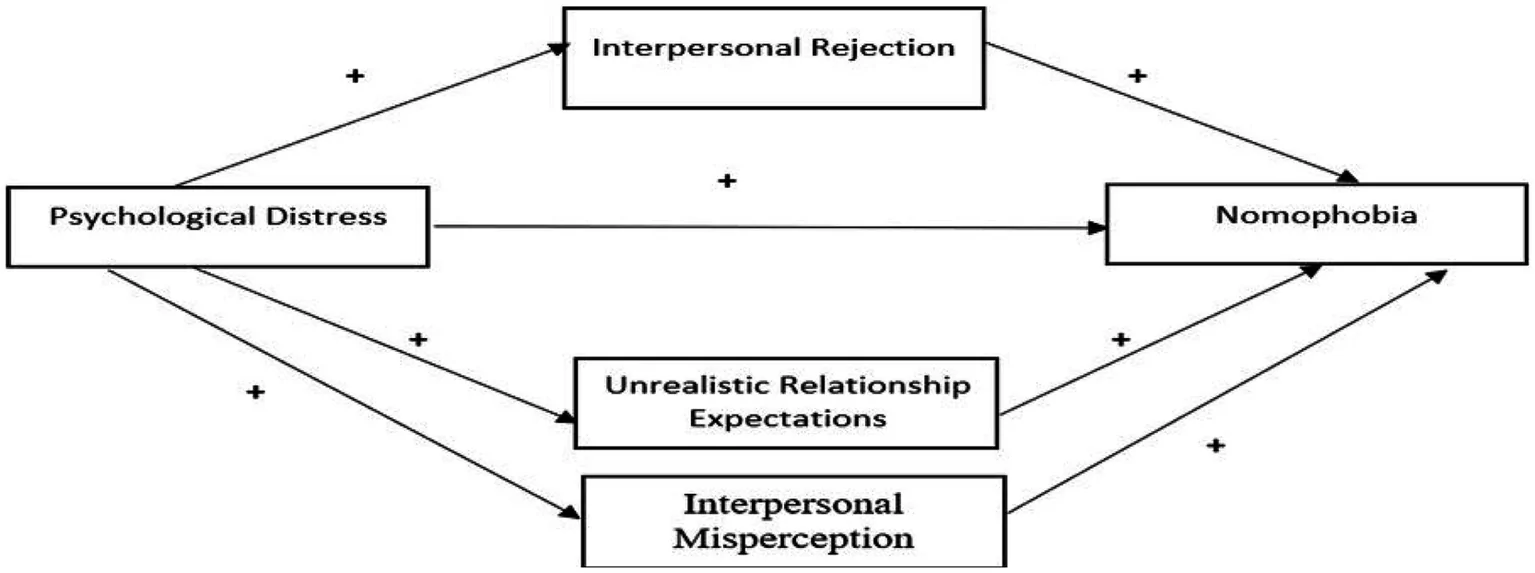
Conceptual model depicting the mediating role of interpersonal cognitive distortions (interpersonal rejection, unrealistic expectations, and misperception) in the relationship between psychological distress and nomophobia.

To our knowledge, no previous study has directly tested the three dimensions of interpersonal cognitive distortions as parallel mediators of the relationship between psychological distress and nomophobia. This gap is important because adolescence is a stage of heightened vulnerability to distorted interpersonal beliefs and digital reassurance-seeking (Twenge and Campbell, 2018). The conceptual framework of the current study is illustrated in Figure 1, which depicts the proposed mediating role of interpersonal cognitive distortions in the relationship between psychological distress and nomophobia.

Therefore, the present study aims to investigate the mediating role of interpersonal cognitive distortions in the relationship between psychological distress and nomophobia. In doing so, it seeks to clarify the underlying psychological mechanisms through which emotional distress and maladaptive thought patterns interact to influence problematic smartphone use. Specifically, the study examines how individuals experiencing higher levels of psychological distress may develop distorted perceptions and expectations in their interpersonal relationships, which in turn may heighten their fear of being disconnected or unavailable through mobile devices. By identifying these pathways, the study provides a more comprehensive understanding of the cognitive and emotional factors contributing to nomophobia and highlights the importance of interventions that simultaneously address emotional regulation and cognitive restructuring to promote healthier technology use.

### Hypotheses

Grounded in the integrated theoretical framework and emphasizing the role of Interpersonal Cognitive Distortions, the following hypotheses are proposed:

*H1*: Psychological distress will significantly and positively predict levels of nomophobia.

*H2*: Psychological distress will significantly predict the dimensions of interpersonal cognitive distortions.

*H3*: The aspects of interpersonal cognitive distortions will significantly predict levels of nomophobia.

*H4:* The relationship between psychological distress and nomophobia will be mediated by the aspects of interpersonal cognitive distortions.

## Materials and methods

### Study design

This cross-sectional study was conducted online with the help of Google Forms between May 2025 and August 2025. Ethical approval was obtained from the Institutional Ethics Committee of the Faculty of Psychology, Aligarh Muslim University, (R.No/1514/PSY). The study was conducted in compliance with the Declaration of Helsinki on Research Involving Human Subjects. As all participants were minors, they were thoroughly briefed on the study’s objectives, procedures, potential risks, and benefits prior to participation. Written informed consent was obtained electronically from their parents or legal guardians, along with assent from the students themselves. Participants were assured of confidentiality, anonymity, and their right to withdraw from the study at any stage without any consequences. To ensure that only target participants completed the survey, the form was distributed exclusively through university student WhatsApp groups. Participants were required to provide their institutional enrolment number or student email to confirm eligibility. Google Forms “limit to one response per account” setting was activated to prevent duplicate entries.

### Participants and sampling

Participants were adolescents enrolled in university-affiliated colleges in Uttar Pradesh, India. In the Indian education system, students may enter pre-university or intermediate courses as early as 15 years of age; therefore, the age range of 15–19 years was selected. *A priori* sample size estimation was conducted using G*Power 3.1, with the test family set to linear multiple regression: fixed model, *R*^2^ deviation from zero. Assuming a medium effect size (*f*^2^ = 0.15), an alpha level of 0.05, and a desired statistical power of 0.95, the minimum required sample size was calculated to be 119 participants. To enhance statistical reliability, generalizability, and to account for potential missing data, a larger sample of 500 students was targeted. This strategy is consistent with recommendations for observational research where variability in response quality is anticipated.

### Measures

*Nomophobia Questionnaire (NMP-Q)*: NMP-Q (Yildirim and Correia, 2015) is a 20-item self-report instrument designed to assess the severity of nomophobia, defined as the fear or anxiety associated with being without access to a mobile phone. Each item is rated on a 7-point Likert scale ranging from 1 (strongly disagree) to 7 (strongly agree), with total scores ranging from 20 to 140. Higher scores indicate greater levels of nomophobia. Score interpretation is as follows: a total score of 20 reflects the absence of nomophobia, scores between 21 and 59 indicate a mild level, scores between 60 and 99 reflect a moderate level, and scores of 100 or above represent a severe level of nomophobia. The NMP-Q has been widely used in research and has demonstrated robust psychometric properties, including high internal consistency and construct validity (Jahrami et al., 2023). In the present study, internal consistency reliability (Cronbach’s alpha) of this measure was found as 0.82.

*Depression Anxiety Stress Scale (DASS-21)*: Psychological distress was measure by using DASS-21 (Lovibond and Lovibond, 1995). This is a 21 item self-report tool used to evaluate the level of stress, anxiety, and depression. Each subscale consists of seven items rated on a 4-point Likert scale that goes from 0 (did not apply to me at all) to 3 (applied to me very much or most of the time). Higher the score on each subscale greater is the symptom severity. Standardized cutoff values, which are different for each subscale, are used to classify severity levels as normal, mild, moderate, severe, or extremely severe. It is a popular tool for evaluating emotional distress in clinical and research contexts, and has undergone extensive validation. Internal consistency reliability (Cronbach’s alpha) of this measure was found as 0.89.

*Interpersonal Cognitive Distortion Scale (ICDS)*: The ICDS (Hamamcı and Büyüköztürk, 2004) a standardized 19-item self-report tool intended to evaluate maladaptive cognitive patterns associated with interpersonal relationships, was used to measure interpersonal cognitive distortions. The three subscales of the ICDS have been empirically validated: Interpersonal Rejection, which measures beliefs about expected rejection or exclusion by others; Unrealistic Relationship Expectations, which measures rigid and idealized assumptions about interpersonal dynamics; and Interpersonal Misperception, which measures the tendency to negatively bias one’s interpretation of the actions and intentions of others. A 5-point Likert scale, with 1 denoting “strongly disagree” and 5 denoting “strongly agree,” is used to rate each item. A composite score is derived by adding all item responses. Higher scores signifies a higher prevalence of distorted interpersonal cognitions. The ICDS has demonstrated robust psychometric properties in clinical (Cronbach *α* = 0.92) and non-clinical sample (Cronbach *α* = 0.93) (Özdel et al., 2014). In this study, reliability was high across subscales (*α* = 0.82–0.88).

*Demographic Questionnaire*: A demographic form collected participant age, gender, and family type (nuclear or joint). In addition, participants reported their average daily time on social media. Response categories were: “less than 30 minutes,” “30 minutes to 1 hour,” “1–2 h,” “2–3 h,” “4–5 h,” and “5 hours or more.” These categories were adapted from previous studies on adolescent digital media use to capture typical patterns (Twenge and Campbell, 2018).

### Procedure

The study was conducted online using Google Forms. The purpose of the study was clearly explained at the beginning of the form. Informed consent was obtained from each participant before proceeding, and confidentiality of their responses was assured. Participation was entirely voluntary. Clear instructions were provided for completing the form. First, demographic information was collected, followed by the administration of the relevant psychological scales. Participants were requested not to skip any questions. Cultural sensitivities were respected throughout the data collection process. All responses were anonymized, and the data were securely stored to ensure confidentiality.

### Statistical analysis

Data analysis was conducted using SPSS version 25.0 and PROCESS Macro version 3.4. Initially, preliminary data screening was performed to identify and address any issues related to missing values, outliers, and data entry errors, ensuring the dataset’s integrity and suitability for subsequent analyses. Descriptive statistics, including means, standard deviations, frequencies, and percentages, were computed using SPSS 25.0 to summarize the demographic characteristics and main study variables. To examine the hypothesized mechanisms through which psychological distress (independent variable) influences nomophobia (dependent variable), a parallel mediation analysis was conducted using PROCESS Macro (Model 4). This model allowed for simultaneous testing of multiple mediators, specifically the dimensions of interpersonal cognitive distortions, to determine their individual and collective mediating effects. The analysis provided estimates of direct, indirect, and total effects, along with bootstrap confidence intervals (typically based on 5,000 resamples) to assess the significance of the mediation pathways (Hayes, 2018). The statistical significance of the indirect effect was determined by 95% Bootstrap CIs that excluded zero. The magnitude of indirect effects was assessed using the completely standardized indirect effect (IE) and R2med (=IE2), following Preacher and Kelley’s recommendations. The effect sizes of 0.01, 0.09, and 0.25 were interpreted as small, medium and large, respectively, (Preacher and Kelley, 2011). This approach enabled a robust examination of the underlying psychological processes linking psychological distress to nomophobia.

## Results

The sample comprised 500 adolescents, who reported using the internet and social media and gave their consent for the study. They were selected randomly from the general population with the age range of 15–19 years (Mean age = 18.15 ± 2.12). Table 1 shows almost half, 51% (*n* = 256) of the participants were male and remaining 244 participants (49%) were female. Three hundred ninety eight adolescents were from nuclear family (79.6%) and remaining 102 were from joint family (20.4%). Of the total participants, 214 used the mobile phone for four to 5 h (42.8%), 145 participants spent 2–3 h on their mobile phone (29%), 131 participants used it for more than 6 h while the remaining 10 used mobile phone for 1–2 h (2%).

**Table 1:** Demographic profile of the participants (*N* = 500).

Variable	*n*	%
Gender		
Male	256	51.0%
Female	244	49.0%
Age (in years)	15–19 years (Mean = 18.15, SD = 2.12)	
Family type		
Nuclear	398	79.6
Joint	102	20.4
Daily mobile phone usage		
1–2 h	10	2.0%
2–3 h	145	29.0%
4–5 h	214	42.8%
More than 6 h	131	26.2%

Table 2 presents the descriptive statistics, reliability estimates, and correlations among the study variables. Participants reported moderate levels of psychological distress on the DASS-21 (M = 25.99, SD = 4.67). Regarding interpersonal cognitive distortions, the means and standard deviations were as follows: interpersonal rejection (M = 20.70, SD = 7.72), unrealistic relationship expectations (M = 15.76, SD = 4.12), and interpersonal misperception (M = 6.95, SD = 2.08). For nomophobia, the mean score was 43.05 (SD = 13.01). All measures demonstrated good internal consistency, with Cronbach’s alpha coefficients ranging from 0.82 to 0.89. Correlational analyses indicated that nomophobia was positively and significantly associated with psychological distress (*r* = 0.302, *p* < 0.001) and with all dimensions of interpersonal cognitive distortion, including interpersonal rejection (*r* = 0.297, *p* < 0.001), unrealistic relationship expectations (*r* = 0.279, *p* < 0.001), and interpersonal misperception (*r* = 0.152, *p* < 0.001).

**Table 2:** Descriptive statistics, reliability estimates, and correlations among study variables.

Variable	1	2	3	4	5	Mean	SD	*α*
1. PYD	–	0.215^**^	0.170^**^	0.208^**^	0.302^**^	25.99	4.67	0.890
2. IPR		–	0.356^**^	0.177^**^	0.297^**^	20.70	7.72	0.822
3. URE			–	0.137^**^	0.279^**^	15.76	4.12	0.827
4. IPM				–	0.152^**^	6.95	2.08	0.880
5. NOP					–	43.05	13.01	0.820

PYD, Psychological Distress; IPR, Interpersonal Rejection; URE, Unrealistic Relationship Expectations; IPM, Interpersonal Misperception; NOP, Nomophobia; *α*, Alpha coefficient; ^**^p < 0.01.

Before conducting the mediation analysis, the important assumptions underlying the multiple regression were checked. There was no multicollinearity, with Variance Inflated factors values ranging from 1 to1.193 (VIF < 10) and Tolerance values were between 0.86 and 0.91 (>0.10). Normality of residuals was evaluated through Q-Q plots, it was approximately normal. The scatterplots of residuals showed linearity and constant variance. Moreover, the Durbin-Watson value of 1.98 indicated that errors were independent (close to 2). Thus, the assumptions for regression were satisfactorily met.

**Figure 2:**
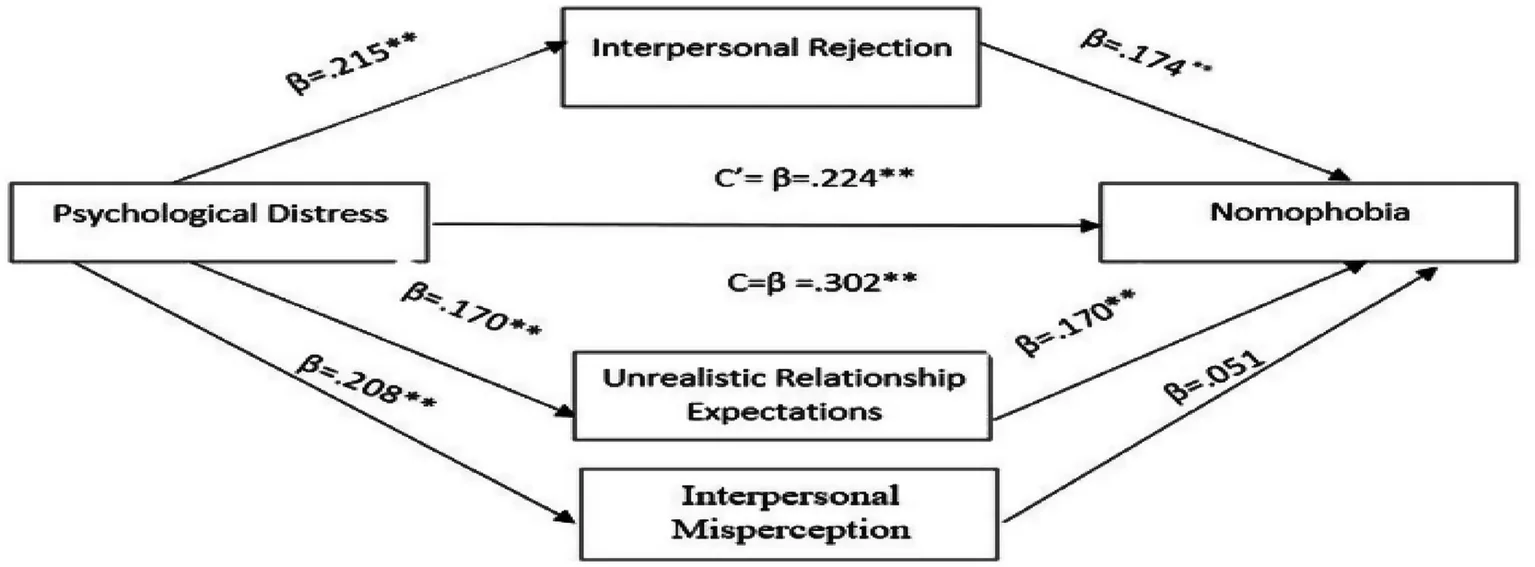
Parallel mediation model illustrating the effects of interpersonal rejection, unrealistic relationship expectations, and interpersonal misperception on the association between psychological distress and nomophobia.

The effects of multiple mediators were examined in a single integrated model using parallel mediation (Process Macro Hayes 3.4 Model 4). Psychological distress was considered as the predictor variable, nomophobia as the criterion variable, and interpersonal cognitive distortions as mediators (interpersonal rejection, unrealistic relationship expectation, and interpersonal misperception). Table 3 summarizes the results of direct and total effects of psychological distress on nomophobia and the indirect effects through the dimensions of cognitive distortions (Figure 2).

**Table 3:** Total direct and indirect effects of psychological distress on nomophobia.

Effects	*B*	*β*	SE	95%CI	
				Lower	Upper
Total effect Psychological Distress (PYD) → Nomophobia (NOF)	0.839	0.302^**^	0.119	0.605	1.027
Direct effect PYD → NOF	0.622	0.224^**^	0.119	0.389	0.855
Indirect effect (total)	0.217	0.078^*^	0.018	0.045	0.114
Interpersonal rejection (INR) PYD → INR → NOF	0.179	0.039^*^	0.013	0.017	0.066
Unrealistic relationship expectation (URE) PYD → URE → NOF	0.170	0.029^*^	0.010	0.011	0.051
Interpersonal misperception (INM) PYD → INM → NOF	0.051	0.011	0.009	−0.007	0.029

*β*, Standardized coefficient; *B*, Unstandardized coefficient; SE, Standard Error; CI, Confidence Interval; ^*^p < 0.005; ^**^p < 0.001.

Table 3 clearly shows that psychological distress significantly and positively predicted nomophobia (*β* = 0.224, *p* < 0.001). In Figure 2, dimensions of interpersonal cognitive distortion—Interpersonal rejection (*β* = 0.215, *p* < 0.001), unrealistic relationship expectations (*β* = 0.170, *p* < 0.001), and interpersonal misperception (*β* = 0.208, *p* < 0.001), were all substantially and positively predicted by psychological distress. In addition, nomophobia was significantly predicted by interpersonal rejection (*β* = 0.174, *p* < 0.001), and unrealistic relationship expectations (*β* = 0.170, *p* < 0.001) facets of interpersonal cognitive distortion, but not by interpersonal misperception (*β* = 0.051, *p* < 0.005). Total effect revealed that nomophobia was significantly and positively predicted by psychological distress (*β* = 0.302, *p* < 0.001).

With regard to mediation, the completely standardized indirect effect shows that, interpersonal rejection significantly statistically mediated the relationship between psychological distress and nomophobia, IE = 0.039, Boot SE = 0.013, 95% CI: [0.017; 0.066,], as zero was excluded from 95% CI. It shows that higher level of psychological distress leads to interpersonal rejection, which in turn causes nomophobia. Similarly, the unrealistic relationship expectations, also significantly mediated this relationship, implying that higher distress was linked to unrealistic expectations that resulted in nomophobia, IE = 0.029, Boot SE = 0.013, 95% CI:[0.011; 0.051]. In contrast, interpersonal misperception did not significantly buffer the association between distress and nomophobia, IE = 0.011, Boot SE = 0.011, 95% CI: [− 0.007; 0.029], because zero lies between the 95% CI. (Table 3).

In addition to testing the statistical significance, the effect size of the indirect paths was also examined. The indirect effect via interpersonal rejection (IE = 0.039) and unrealistic relationship expectations (IE = 0.029) were statistically significant but small in magnitude, explaining approximately 0.15 and 0.04% of the variance in nomophobia, respectively. In sum, the partial mediation was supported, the practical contribution of the significant mediators was modest.

## Discussion

This study provides preliminary evidence suggesting that psychological distress may serve as a precursor to nomophobia. While existing literature has often examined nomophobia as a predictor of distress, positioning it as a cause of anxiety or depression (Rawas et al., 2021; Abualruz et al., 2024; Demirci et al., 2015), the present research approached the relationship from the opposite direction, investigating psychological distress as a predictor of nomophobia. This perspective broadens understanding by recognizing that emotional vulnerability can heighten adolescents dependence on smartphones. The relationship, however, appears complex and is likely influenced by psychological as well as cognitive factors. Grounded in Uses and Gratifications Theory, Compensatory Internet Use Theory, and the Matthew Effect, this study examined how distress may contribute to nomophobia through maladaptive cognitive pathways.

The results revealed a positive association between psychological distress and nomophobia, suggesting that adolescents experiencing psychological distress may turn to mobile phones as a means of regulating emotions and maintaining social connections during difficult life phases. This finding is consistent with Uses and Gratifications Theory, which emphasizes that individuals actively choose media to satisfy cognitive, affective, integrative, and social needs (Katz et al., 1974). However, while smartphones provide temporary comfort, excessive reliance can escalate into nomophobia, reinforcing dependence rather than alleviating distress (Elhai et al., 2020). These results support H1, indicating that adolescents with higher distress levels are more likely to develop nomophobia.

The findings also highlight the role of cognitions in coping behavior. To the best of our knowledge, the mediating role of ICD’s in nomophobia has not been empirically tested prior to this study. Previous research has used mediation and moderation models to identify several psychological and interpersonal mechanisms underlying nomophobia, including loneliness, attachment, online social interaction, and social support (Sun et al., 2024; Khan et al., 2025). However, the specific role of interpersonal cognitive distortions as mediating mechanisms between psychological distress and nomophobia has not been directly examined. Our results showed that psychological distress was positively related to ICD’s. These findings confirm H2, indicating that psychological distress significantly predicted interpersonal cognitive distortions, Further Nomophobia was associated with two specific ICDs, interpersonal rejection and unrealistic relationship expectations. However, interpersonal misperception did not strongly predict nomophobia. Thus, H3 was partially supported, as nomophobia was significantly predicted by interpersonal rejection and unrealistic relationship expectations, but not by interpersonal misperception. A likely explanation is that while misperceptions of others intentions can heighten social discomfort, they may not be sufficient to drive smartphone overuse unless accompanied by more salient distortions, such as fear of rejection or rigid interpersonal expectations. Adolescents may misinterpret others behavior but not necessarily feel compelled to compensate digitally unless they also anticipate rejection or demand constant responsiveness. This interpretation is consistent with prior findings showing that rejection sensitivity, rather than general misperceptions, is more predictive of problematic smartphone use. (Li et al., 2023; O’Day and Heimberg, 2021).

The results also clarify why H4 was only partially supported. While psychological distress significantly predicted all three ICD’s, only interpersonal rejection and unrealistic expectations modestly mediated the distress and nomophobia pathway. These findings suggest that certain distortions exert greater influence in translating distress into digital reliance. Adolescents who expect rejection may turn to smartphones for reassurance, while those with unrealistic relationship standards may idealize digital interactions, expecting constant availability and emotional responsiveness. By contrast, misperceptions alone may not create a strong enough motive for smartphone dependence. From a cognitive-behavioral perspective, the differential effects of the three ICD dimensions may reflect differences in how specific cognitions translate psychological distress into behavioral responses. Interpersonal rejection may increase fear of exclusion and the need for reassurance, leading distressed adolescents to use smartphones to maintain social connection, monitor responses, and reduce interpersonal uncertainty. Similarly, unrealistic relationship expectations may create dissatisfaction when actual responsiveness does not match expectations, encouraging repeated digital communication and checking for reassurance. These processes may reinforce smartphone reliance as a short-term coping strategy, consistent with Compensatory Internet Use Theory (Kardefelt-Winther, 2014). In contrast, interpersonal misperception may increase social uncertainty or discomfort without necessarily producing the same motivation for reassurance-seeking or continuous smartphone engagement. Thus, although misperception may represent a cognitive vulnerability, its behavioral connection with nomophobia may be weaker or more indirect. These differences may also be particularly relevant during adolescence, a developmental period characterized by heightened sensitivity to peer acceptance, social evaluation, and interpersonal feedback (Twenge and Campbell, 2018). Consequently, rejection-related concerns and expectations of interpersonal responsiveness may have greater influence on smartphone dependence than interpersonal misperception alone. However, these interpretations remain theoretical because the cross-sectional design does not establish causal or temporal relationships among psychological distress, ICDs, and nomophobia.

Comparison with previous mediation and moderation studies further clarifies the contribution of the present findings. For instance, Sun et al. (2024) found that loneliness and attachment mediated the link between neuroticism and nomophobia, while Khan et al. (2025) demonstrated that online social interaction mediated the effect of loneliness on nomophobia. These studies indicate that nomophobia may develop through affective and interpersonal pathways, but they do not address whether maladaptive beliefs about interpersonal relationships explain the association between psychological distress and nomophobia. Similarly, Karaoglan Yilmaz et al. (2024) reported associations between nomophobia, depression, and social anxiety among college students. These findings highlight the importance of emotional vulnerability in understanding nomophobia, whereas the present study extends this perspective by examining the cognitive mechanisms through which psychological distress may be associated with nomophobia. Moreover, Lu and Yeo (2015) identified cognitive distortions as predictors of pathological internet use, underscoring the importance of maladaptive cognitions in digital dependence. Taken together, these studies suggest that nomophobia is influenced by multiple affective, interpersonal, and cognitive factors. The current findings extend this literature by identifying interpersonal rejection and unrealistic relationship expectations as specific cognitive pathways linking psychological distress to nomophobia among adolescents. Thus, the contribution of the present study is not simply the use of a mediation model, but the examination of a distinct interpersonal-cognitive pathway that has received limited empirical attention in nomophobia research. The differential findings across the ICD dimensions further suggest that the specific content of interpersonal cognition may determine its behavioral relevance to nomophobia.

The present findings can also be interpreted in light of recent research examining nomophobia within broader patterns of digital dependence and digital wellbeing. Durmuş Sarıkahya et al. (2026) found that nomophobia mediated the relationship between digital game addiction and fatigue, while Netless phobia moderated this association, suggesting that digital-related fears may contribute to functional consequences of excessive digital engagement. Similarly, Özbay et al. (2026) reported that students who engaged in digital detox practices exhibited lower levels of nomophobia and Netless phobia and higher academic motivation, self-efficacy, and academic performance. Although these studies examined different populations and outcomes from the present investigation, they reinforce the view that nomophobia is embedded within a wider digital-behavioral context and may be associated with meaningful psychological and functional consequences. Whereas these recent studies emphasize behavioral and digital-contextual mechanisms, and earlier mediation studies have emphasized loneliness, attachment, and online social interaction, the present study identifies an interpersonal-cognitive pathway linking psychological distress to nomophobia. Specifically, our findings suggest that interpersonal rejection and unrealistic relationship expectations may represent cognitive mechanisms through which emotional distress is associated with greater smartphone dependence among adolescents. This provides a complementary explanation of nomophobia that extends existing affective, behavioral, and interpersonal models.

This study makes several important contributions to the understanding of nomophobia and its psychological underpinnings. First, it extends previous mediation and moderation research on nomophobia by introducing interpersonal cognitive distortions as a distinct cognitive pathway linking psychological distress with nomophobia. Whereas previous models have emphasized loneliness, attachment, online social interaction, social support, personality, or related psychological vulnerabilities, the present study examines whether specific maladaptive beliefs about interpersonal relationships explain this association. In particular, unrealistic relationship expectations and heightened sensitivity to interpersonal rejection were identified as particularly influential, suggesting that not all cognitive distortions exert equal effects on smartphone dependence. This differential pattern provides a more nuanced cognitive-behavioral interpretation of nomophobia by suggesting that interpersonal cognitions directly related to rejection and social responsiveness may be more relevant to smartphone dependence than interpersonal misperception alone. These findings underscore the importance of designing interventions that specifically target distorted interpersonal beliefs, rather than relying on generic approaches, in order to more effectively mitigate maladaptive patterns of smartphone use. Second, the study’s focus on adolescents—an age group especially sensitive to peer evaluation and interpersonal insecurities—offers valuable insights for prevention and intervention efforts. Adolescents are in a critical developmental stage, characterized by heightened social awareness and vulnerability to peer-related stressors, making them particularly susceptible to both cognitive distortions and excessive smartphone dependence. Interventions for this population may benefit from incorporating cognitive-behavioral strategies aimed at identifying and restructuring maladaptive interpersonal beliefs, while simultaneously fostering adaptive coping mechanisms and resilience. By targeting the specific cognitive pathways that link distress to nomophobia, such interventions may more effectively reduce the risk of problematic smartphone use and support healthier social and emotional development.

Several limitations of the present study should be acknowledged. First, the cross-sectional design precludes any causal inferences, meaning that the observed associations between psychological distress, interpersonal cognitive distortions, and nomophobia should be interpreted as preliminary rather than definitive. Longitudinal or experimental research designs are needed to determine the directionality of these relationships and to assess potential bidirectional influences between distress and problematic smartphone use. Second, the reliance on self-report measures may have introduced response biases, including social desirability or perceptual distortions. Future research could benefit from incorporating objective behavioral measures of smartphone use, ecological momentary assessments, or informant reports to provide a more robust and multi-faceted understanding of the phenomena. Third, the study sample was restricted to Indian adolescents, which may limit the generalizability of the findings to other cultural, age, or demographic groups. Cross-cultural studies are warranted to examine whether these patterns of associations hold in different sociocultural contexts and to explore potential moderating effects of cultural norms, family dynamics, or educational environments. Finally, future studies could expand the scope of investigation by including additional psychological and social variables that may interact with interpersonal cognitive distortions in predicting digital dependence. Constructs such as attachment style, fear of missing out, or resilience may provide important insights into individual differences in susceptibility to nomophobia and could inform more targeted prevention and intervention strategies. Future research should also directly compare the interpersonal-cognitive pathway identified in the present study with previously established mediators such as loneliness, attachment, and online social interaction, preferably using longitudinal designs to determine whether these mechanisms operate independently or sequentially. Collectively, addressing these limitations would strengthen the evidence base and guide the development of more effective approaches to managing problematic smartphone use among adolescents.

## Conclusion

In conclusion, this study suggests that psychological distress may contribute to nomophobia both directly and indirectly, with interpersonal rejection and unrealistic relationship expectations serving as mediators. Interpersonal misperception, however, did not play a significant role, highlighting that not all cognitive distortions equally predict digital reliance. These findings extend previous mediation and moderation research on nomophobia by positioning specific interpersonal cognitions as a distinct mechanism in the distress–nomophobia pathway, particularly in adolescence. Rather than suggesting that mediation itself is novel, the present study contributes by identifying interpersonal rejection and unrealistic relationship expectations as previously underexamined cognitive pathways linking psychological distress with nomophobia. Although preliminary, the results underscore the importance of addressing both emotional and cognitive vulnerabilities in interventions. By targeting maladaptive interpersonal beliefs through therapeutic strategies, it may be possible to reduce excessive smartphone reliance and mitigate the risk of nomophobia. Future longitudinal and cross-cultural research will be critical for confirming these pathways and refining interventions. Overall, this study provides important insights into the psychological and interpersonal factors that make adolescents more susceptible to nomophobia, offering directions for both theory and practice in addressing this emerging concern.

## Data Availability

The original contributions presented in the study are included in the article/supplementary material, further inquiries can be directed to the corresponding author/s.
